# The innominate artery, a seemingly benign artery that can cause major airway complications

**DOI:** 10.5339/qmj.2024.qitc.17

**Published:** 2024-03-26

**Authors:** Safa Mahgoub, Manu Sundaram, Andrew Durward

**Affiliations:** Division of Pediatric Critical Care, Department of Pediatrics, Sidra Medicine, Ar-Rayyan, Qatar Email: smahgoub1@sidra.org; Division of Cardiac Intensive Care, Department of Pediatrics, Sidra Medicine, Ar-Rayyan, Qatar

**Keywords:** Innominate artery, tracheal compression, tracheo-innominate fistula

## Introduction

The innominate artery is the first branch of the aortic arch. It traverses anterior to the trachea and crosses it at the 9th tracheal ring from left to right without airway compression.^1^ In rare cases, due to anatomical variation, the innominate artery can exert severe compression on the trachea,^2^ which in tracheostomized patients can contribute to the development of tracheo-innominate fistulas that lead to life-threatening massive bleeding.^3^

## Cases Presentation

**Figure 1.**
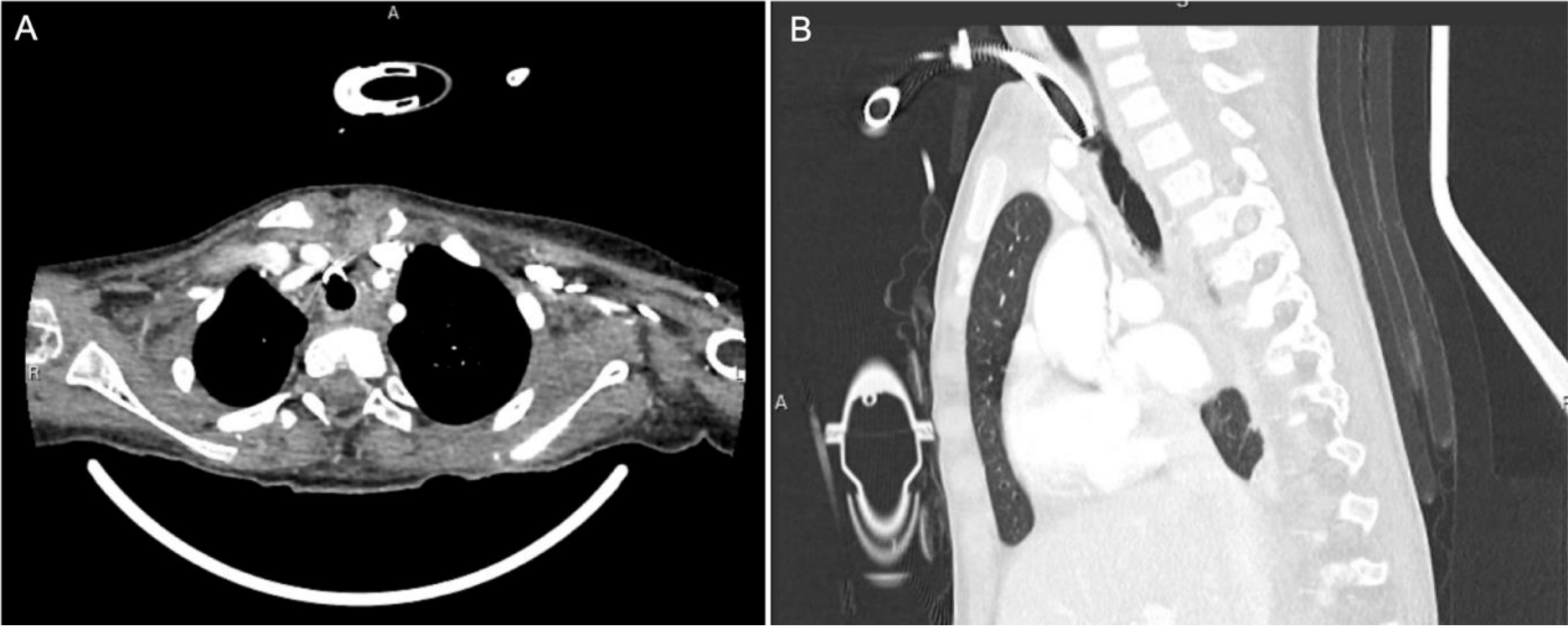
CT chest with contrast showing the close proximity between the tip of the tracheostomy tube and the innominate artery. (A) Axial plane. (B) Sagittal plane.

Here we present two cases in which innominate artery compression of the trachea caused life-threatening airway problems. The first case is a 4-year-old girl who was newly tracheostomized following a traumatic cervical spinal injury. She developed acute intratracheal bleeding due to erosion of the innominate artery into the trachea with a cuffed tracheostomy tube, creating a tracheo-innominate fistula. Despite prompt ENT and cardiothoracic surgical intervention, this progressed to massive life-threating hemorrhage, with hypovolemic shock resulting in prolonged CPR and hypoxic ischemic brain injury. A retrospective review of her initial chest CT revealed close proximity between the end of her tracheostomy tube tip and the innominate artery (Figure 1), which is a known risk factor for tracheo-innominate fistula formation.^1^

**Figure 2.**
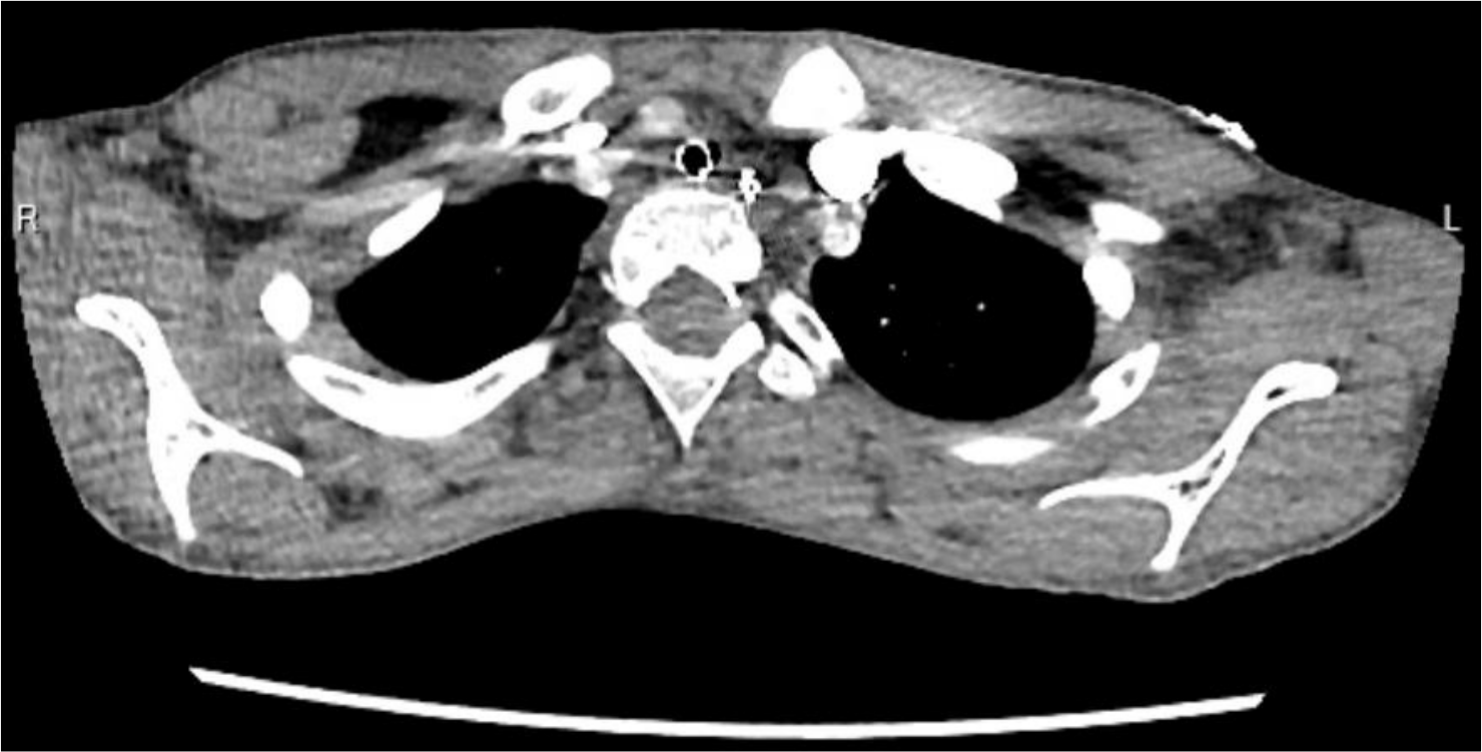
CT chest with contrast showing the innominate artery abuts the trachea causing focal compression with no air seen around the endotracheal tube.

The second case is a 9-year-old boy with dystonia and a history of multiple cardiorespiratory arrests. CT revealed that the cause was a narrow thoracic inlet, where the trachea was compressed by the innominate artery between the sternum and vertebral bodies (Figure 2). Airway compression was relieved by an aortopexy and sternal expansion. The child was extubated and is recovering in PICU.

## Conclusion

These cases illustrate the important, although rare, life-threatening complications of tracheal compression by the innominate artery under certain conditions. Clinicians should be aware of these complications and consider them in the differential of patients with airway problems.

## Conflict of Interest

All authors do not have financial or any other relevant relationships with commercial interest to disclose.
